# Metabolomic effects of androgen deprivation therapy treatment for prostate cancer

**DOI:** 10.1002/cam4.3016

**Published:** 2020-03-31

**Authors:** Jen‐Tsan Chi, Pao‐Hwa Lin, Vladimir Tolstikov, Taofik Oyekunle, Emily Y. Chen, Valerie Bussberg, Bennett Greenwood, Rangaprasad Sarangarajan, Niven R. Narain, Michael A. Kiebish, Stephen J. Freedland

**Affiliations:** ^1^ Department of Molecular Genetics and Microbiology Center for Genomics and Computational Biology Duke University Medical Center Durham NC USA; ^2^ Department of Medicine Division of Nephrology Duke University Medical Center Durham NC USA; ^3^ BERG LLC Framingham MA USA; ^4^ Duke Cancer Institute Duke University Medical Center Durham NC USA; ^5^ Center for Integrated Research in Cancer and Lifestyle Cedars‐Sinai Los Angeles CA USA; ^6^ Durham VA Medical Center Durham NC USA

**Keywords:** 3‐hydroxybutyric acid, 3‐formyl indole, ADT, androgen sulfate, indole‐3‐carboxaldehyde, ketogenesis, lipidomics, metabolomics, prostate cancer

## Abstract

Androgen deprivation therapy (ADT) is the main treatment strategy for men with metastatic prostate cancer (PC). However, ADT is associated with various metabolic disturbances, including impaired glucose tolerance, insulin resistance and weight gain, increasing risk of diabetes and cardiovascular death. Much remains unknown about the metabolic pathways and disturbances altered by ADT and the mechanisms. We assessed the metabolomic effects of ADT in the serum of 20 men receiving ADT. Sera collected before (baseline), 3 and 6 months after initiation of ADT was used for the metabolomics and lipidomics analyses. The ADT‐associated metabolic changes were identified by univariable and multivariable statistical analysis, ANOVA, and Pearson correlation. We found multiple key changes. First, ADT treatments reduced the steroid synthesis as reflected by the lower androgen sulfate and other steroid hormones. Greater androgen reduction was correlated with higher serum glucose levels, supporting the diabetogenic role of ADT. Second, ADT consistently decreased the 3‐hydroxybutyric acid and ketogenesis. Third, many acyl‐carnitines were reduced, indicating the effects on the fatty acid metabolism. Fourth, ADT was associated with a corresponding reduction in 3‐formyl indole (a.k.a. indole‐3‐carboxaldehyde), a microbiota‐derived metabolite from the dietary tryptophan. Indole‐3‐carboxaldehyde is an agonist for the aryl hydrocarbon receptor and regulates the mucosal reactivity and inflammation. Together, these ADT‐associated metabolomic analyses identified reduction in steroid synthesis and ketogenesis as prominent features, suggesting therapeutic potential of restricted ketogenic diets, though this requires formal testing. ADT may also impact the microbial production of indoles related to the immune pathways. Future research is needed to determine the functional impact and underlying mechanisms to prevent ADT‐linked comorbidities and diabetes risk.

## Graphical abstract

ADT is standard therapy for advanced and metastatic PC with negative metabolic consequences. We determined the metabolomic effects of ADT during the PC treatments. Such analysis identify the unexpected reduced ketogenesis and microbiome‐associated metabolites during ADT

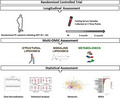

## 1 INTRODUCTION

Androgen deprivation therapy (ADT) is standard therapy for advanced and metastatic prostate cancer (PC).^1^ Approximately one in three men with PC in the US receives ADT.^2^ While ADT is a very effective anticancer treatment, it is unfortunately associated with significant side effects, including decreased libido, impotence, fatigue, osteoporosis, hot flushes, and loss of muscle mass. Other prominent side effects include the metabolic disturbances such as impaired glucose tolerance, insulin resistance, and weight gain, putting men at increased risk for diabetes and cardiovascular (CV) death.^3^ The adverse effect of ADT is further compounded by the fact that most men receiving ADT already have a high prevalence of CV risk factors.^4^ Among nondiabetic men beginning ADT, within 12 weeks, whole‐body insulin sensitivity index decreased by 11.0 ± 8%, insulin resistance by homeostatic model assessment (HOMA) increased by 12.9 ± 5.8%, and fasting plasma insulin increased by 26 ± 9%.^5^ This translated to a 40% increased diabetes risk,^6^ and worsening the glucose control among diabetics on ADT.^7^ Even when ADT was administered for a limited time, such as adjuvant to radiotherapy, diabetes risk still increased.^8^ Beyond increasing the diabetes risk, ADT also promotes increases in fat mass, elevated triglyceride, and LDL cholesterol.^5^ However, the biochemical pathways underlying the above clinical disturbances are not clear.

Various genomic, proteomic, and metabolomic approaches have been employed to dissect the tumor heterogeneity, various clinical courses, and identify novel tumor biomarkers. To understand the metabolic effects of ADT and identify potential biomarkers for treatment monitoring, metabolomic approach using NMR or mass spectrometry can profile small molecule metabolites in the tumor cells, tissues, serum, and urine. In one aspect, metabolomics and all metabolites can serve as an integrated read‐out of all the upstream biochemical activities resulting from the somatic mutations, altered gene expression, and proteome during tumor development and treatments. For example, the integrative analysis of the metabolomic profiling of human tumors with the somatic mutations and gene expression can help to identify the potential genetic drivers of the metabolic changes.^9^, ^10^ When applied to PC, metabolomic analysis reveals several dysregulated metabolic pathways, including sarcosine‐related pathways.^11^, ^12^ In addition, the metabolomic analysis of the serum identified metabolites which can predict the disease progression and tumor aggressiveness.^13^ Other metabolomic studies aim to understand the metabolic dysregulation during PC oncogenesis to identify therapeutic targets.^14^, ^15^, ^16^ While metabolomic approaches may allow us to understand the PC’s response to ADT, only two previous studies, to our knowledge, have determined the effects of ADT on the serum metabolome.^17^, ^18^ One study performed the serum metabolomics for men before and 3 months after ADT.^18^ The study identified 56 significantly altered metabolites that included steroid, bile acid metabolites, and lipid beta‐ and omega‐oxidation.^18^ In the second study, seven serum metabolites were found to be significantly different between the untreated PC patients and healthy controls. Furthermore, among PC patients who did not develop castration resistant PC for at least 2 years, the levels of these metabolites reverted to control levels during the hormonal therapy.^17^ However, the metabolite levels remained abnormal in the PC patients who were not responsive to the hormonal therapies. Therefore, these metabolites may serve as biomarkers predictive of therapeutic response to hormonal therapy. However, much remains unknown about how ADT affects the metabolomic and lipidomic composition of sera. In this study, we performed metabolomic and lipidomic profiling in serum before and after 6 months of ADT treatments. In parallel, we have also integrated the metabolomic data with the ADT‐affected clinical blood chemistry to determine the corresponding changes in glucose, blood lipids, and other measurements of metabolic fitness. This is the first study to perform the lipidomic assays on ADT‐treated men and the first study to correlate metabolomic data to blood chemistry values in men treated with ADT.

## 2 PATIENTS AND METHODS

### 2.1 Study Design

The current study used banked sera samples from men previously enrolled on a prospective randomized phase II trial of an extreme low‐carbohydrate diet (LCD) plus walking intervention for men initiating ADT vs control (asked to make no changes in diet or exercise). The primary results of the study have previously been reported.^19^ In this manuscript, we report the effects of ADT on the serum metabolomics in men randomized to the control arm. The results from the intervention arm will be reported in a future manuscript.

### 2.2 Study participants

In brief, after obtaining IRB approval at each site (Duke University, Durham Veterans Affairs Medical Center [VAMC], and Greater Los Angeles VAMC), men initiating ADT were approached and if they agreed, they signed a written consent that included future analyses. Clinical data and fasting serum samples were collected at baseline, 3, and 6 months post randomization.

Key eligibility included men about to begin hormonal therapy (LHRH agonist, LHRH‐antagonist, or orchiectomy) for PC with an anticipated duration of ≥ 6 months, BMI ≥ 24 kg/m^2^, and phone access to speak with the dietitian. Key exclusion criteria included medication‐controlled diabetes, taking any medications that may interfere with insulin, already consuming a LCD, being vegetarian/vegan, or baseline screening HbA1c > 7%. A total of 42 eligible participants enrolled and were randomized. A total of 40 participants completed the baseline visit (N = 19 LCD, N = 21 control). In the control arm, 20 participants completed the 6‐month visit and the results of the metabolomics profiling from their blood specimen were included in this report.

### 2.3 Data collection and analysis

At each visit of the subject in the study, weight (without shoes and in light clothing) and height were measured, fasting blood collected, and study‐related adverse events were assessed. Fasting blood was analyzed for insulin, glucose, prostate specific antigen (PSA), lipids, and high sensitivity C‐reactive protein (hsCRP). PSA, glucose, and lipids were measured by commercial laboratories (LabCorp and Greater LA VAMC clinical lab). Insulin was measured in an electro‐chemiluminescent immunoassay using an SI‐2400 imager and assay kits from Meso Scale Discovery (Rockville, MD) by Duke Immunoassay Laboratory. The HOMA‐IR was calculated using the approximation (glucose*insulin)/22.5.

Fasting blood samples collected at baseline (BL), 3 months (M3), and 6 months (M6) were used for the metabolomics analysis utilizing GC/MS‐TOF (Gas chromatography–Mass Spectrometry Time of Flight analyzer), QqQ LC (Liquid Chromatography)‐HILIC (Hydrophilic interaction chromatography)‐MS/MS, and TripleTOF LC‐RP‐MS as described previously.^20^ Lipidomics analysis using SCIEX 5600 + MS/MS workflows were utilized to quantify the chemical diversity of the metabolome and lipidome.^20^, ^21^, ^22^

### 2.4 Statistical analysis

Volcano plot was used to visually examine the changes in metabolites at 3 and 6 months from baseline. Significant changes were examined by ANOVA and shown in heatmaps and box plots. Impacted metabolic pathways were selected and mapped using MetaboAnalyst 4.0 (https://www.metaboanalyst.ca/).^23^ Pearson correlation was conducted to examine associations between the clinical variables and metabolites.

## 3 RESULTS

**Figure 1:**
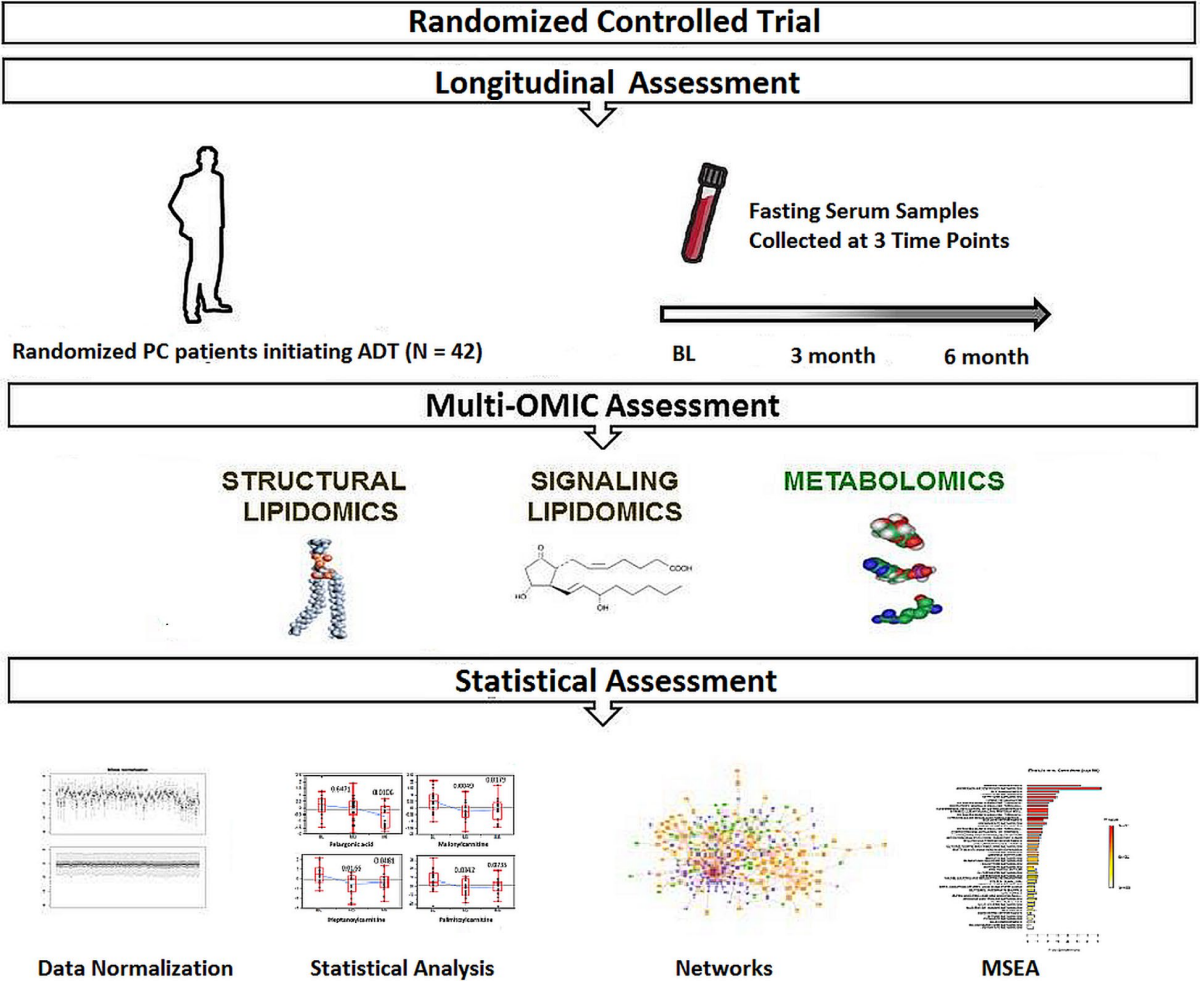
The overview of the sample cohort, the multi‐omics assessment of the serum metabolites and statistical evaluation of the ADT‐induced metabolomic changes. A total of 42 patients with PC were randomized to either ADT alone or ADT combined with low‐carbohydrate diet (LCD) and walking advice intervention for 6 months. Here, we only present the changes of serum metabolomes of 20 in the control arm (20 men) who have completed the 3 and 6 month visits. Fasting serum samples were collected from 20 at baseline (BL), then 3 and 6 months after the initiation of ADT. The serum samples were then subject to the multi‐omic assessment to determine the metabolomic changes, followed by the statistical assessment to identify the ADT‐affected metabolites and metabolic pathways

Over 450 metabolites and 104 signaling lipid species were measured in serum of subjects before and after ADT (Figure 1), and key findings are summarized below.

### 3.1 Serum metabolites altered after 3 months of ADT (M3 metabolites)

**Figure 2:**
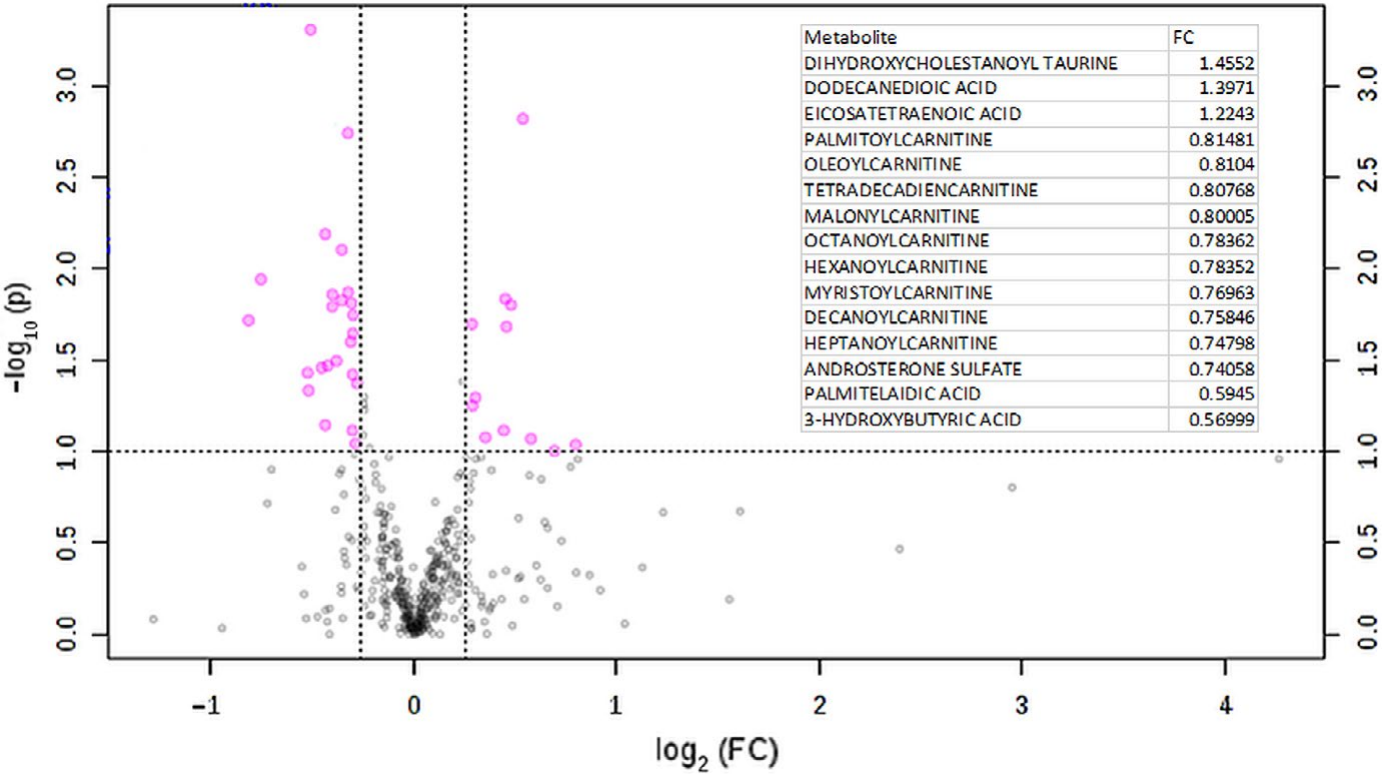
Top Serum Metabolites Altered by 3 months of ADT Treatments. The indicated metabolites have been selected by Volcano analysis of pairwise comparison between the baseline and 3 months ADT treatments. Fold change (FC) for selected metabolites listed in the embedded table

To identify the ADT‐induced metabolomic changes, we first used the univariate statistics to detect differences and volcano plots to visualize the different levels of selected metabolites between baseline (BL, before ADT) and after 3 months (M3) of ADT. The ADT‐affected metabolites were identified based on the thresholds of 1.2‐fold change and t tests *P* < .1. The top ADT‐affected metabolites were shown in Volcano plots along with the metabolites that were significantly changed in a table (Figure 2). At 3 months, the top ADT‐affected metabolites include the reduction of many long‐chain acyl‐carnitines, including hydroxymyristoyl‐carnitine, malonyl‐carnitine, hexanoyl‐carnitine and dodecenoyl‐carnitine, octanoyl‐carnitine and oleoyl‐carnitine, and decanoyl‐carnitines. In addition, ADT significantly reduced the levels of androsterone sulfate, 3‐hydroxyburtirc acid, and indoleacetic acid (IAA), a trypan‐derived metabolite from microbiomes.^23^ In addition, 3 months of ADT (M3) also increased the level of dihydroxycholestanoyl taurine, dodecanedioic acid, and eicosatetraenoic acid.

### 3.2 Serum metabolites altered after 6 months of ADT (M6 metabolites)

**Figure 3:**
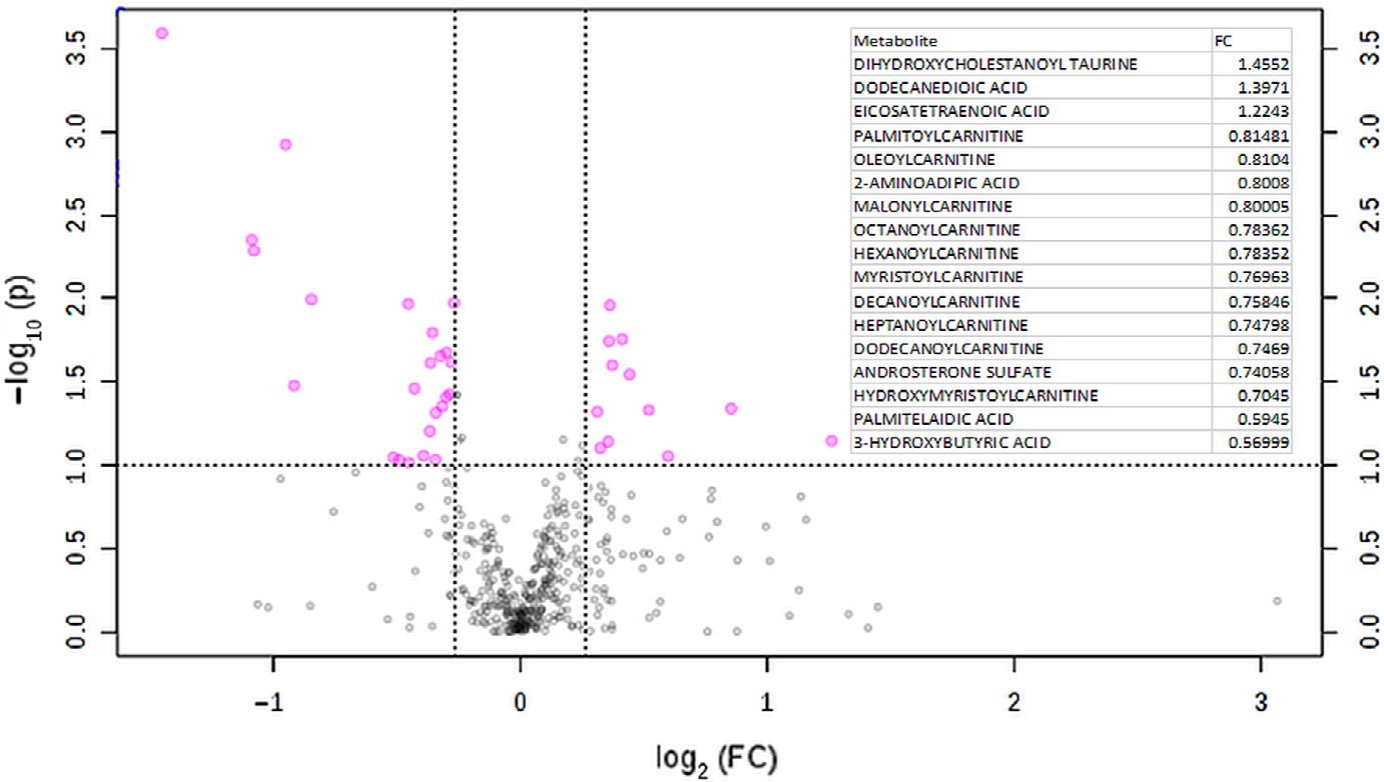
Top Serum Metabolites Altered by 6 months of ADT Treatments. The indicated metabolites have been selected by Volcano analysis of pairwise comparison between the baseline and 6 months ADT treatments. Fold change (FC) for selected metabolites listed in the embedded table

At 6 months, the top ADT‐affected metabolites included the increase in the level of dihydroxycholestanoyl taurine, AMP, N‐acetyl‐glucosamine‐1‐phosphate, mevalonate‐5‐phosphate,and 2‐hydro‐D‐gluconate (Figure 3). In addition, there was also a reduction in several long‐chain acylcarnitines, including malonylcarnitine, oleoylcarnitine, hexanoylcarnitine, tetradecendoycarnitine, heptanoylcarnitine palmitoylcarnitine, decanoyl‐carnitines, and myristolylcarntine. Consistent with the changes at 3 months, ADT at 6 months significantly reduced the levels of androsterone sulfate and 3‐hydroxyburtirc acid. In addition, 6 months of ADT (M6) also reduced the plasma level of organic acids, including oxalic acid, glycolic acid, and nonanoic acid.

### 3.3 Lipidomic analysis

In addition, we also performed the structural lipid analysis which quantified over 1000 lipid molecular species from over 18 lipid classes. Comparison of ADT‐treated vs non‐treated PC patients revealed 26 lipid molecular species at 3 months (Figure S1A) and 24 lipid molecular species at 6 months (Figure S1B) that were statistically significant between the two groups (Tables S1 and S2). Further analysis via ANOVA revealed no significant lipids between the ADT treatment group and nontreatment group (Figure S1C), suggesting that ADT does not impact the serum lipidome in PC patients compared to the effect determined in the metabolome.

### 3.4 Metabolites consistently altered in both M3 and M6

**Figure 4:**
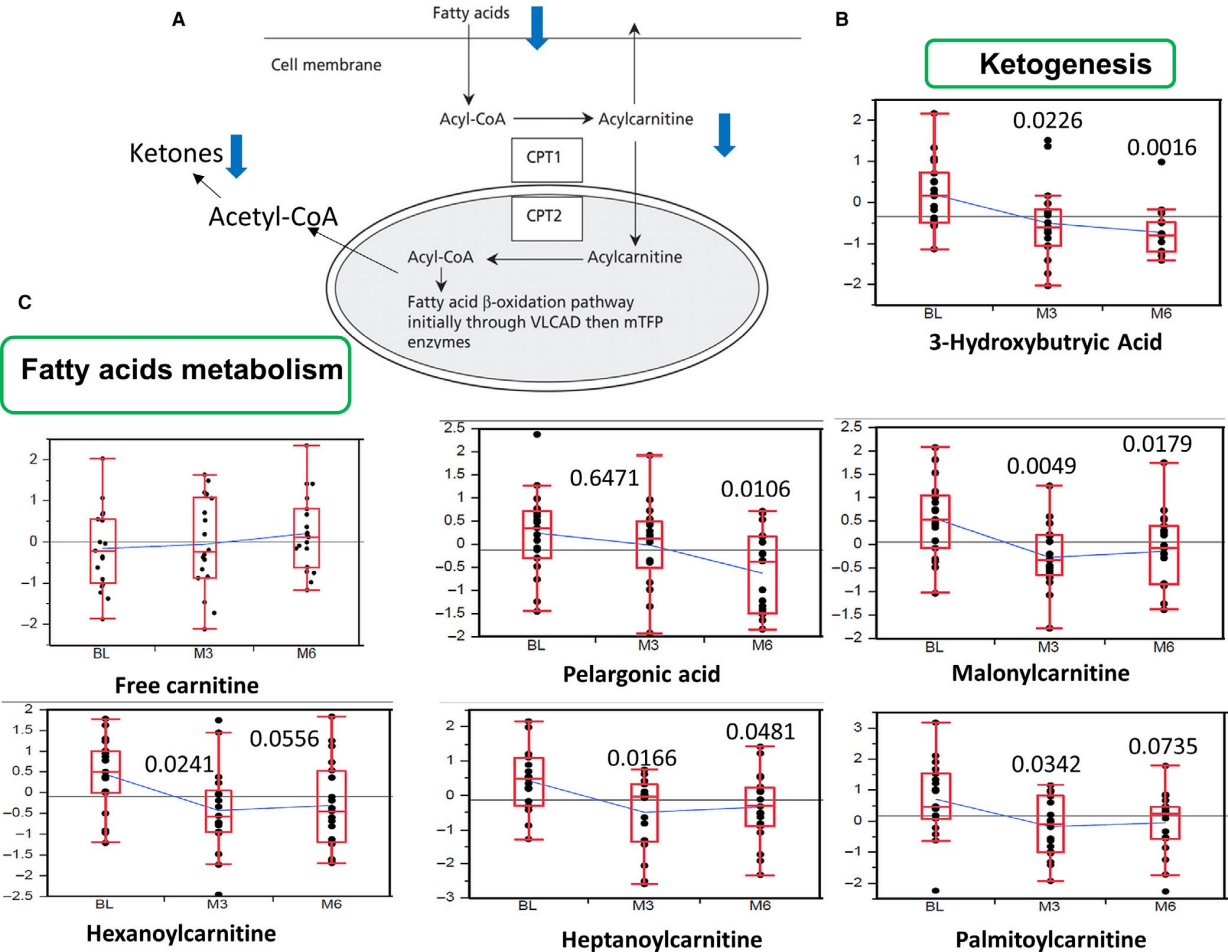
ADT Reduced Metabolites in Ketogenesis and Fatty Acid Metabolism. (A) The metabolic pathway of fatty acid, acylcarnitine and ketones in the context of cellular metabolisms in the cytosols and mitochondria. (B, C) ADT reduced the levels of 3‐hydroxybutryic acid (B), fatty acid (palmitoleic acid), and acylcarnitine (octyanoyl‐carnitine, palmitoyl‐carnitine) (C) without changing the levels of free carnitine. The statistical significance (*P* values) of ADT‐induced changes of indicated metabolites is indicated

In order to identify consistently affected metabolites we applied ANOVA––Simultaneous Component Analysis (ASCA module, MetaboAnlyst 4.0) allowing us to identify the major patterns associated with the time points and phenotype. Several metabolites consistently affected by ADT were in the fatty acid metabolism (Figure 4A). Especially noted is the consistent reduction in the 3‐hydroxybutyric acid (Figure 4B) as well as multiple metabolites in the fatty acid metabolisms (Figure 4C), including a decrease in several long‐chain acyl‐carnitines (malonylcarnitine, heptanoylcarnitine, palmitoylcarnitine, pelargonic acid, and hexanoylcarnitine). However, there was no change in the free carnitine (Figure 4C). These changes consistently reflect the potential impact of ADT on ketogenesis and fatty acids metabolism as shown in Figure 4A.

### 3.5 Pathway analysis of the affected metabolites

**Figure 5:**
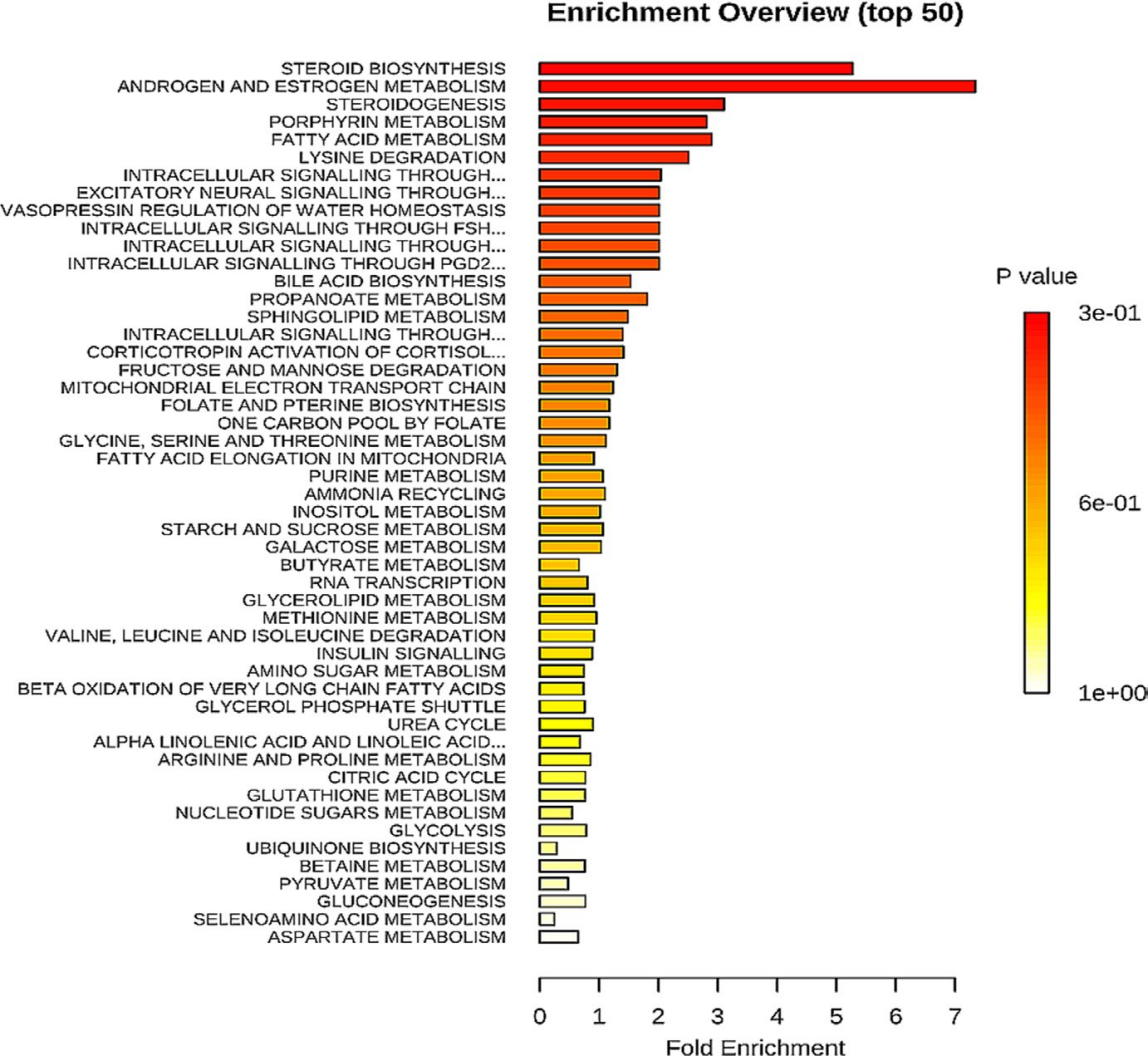
Pathways Enrichment Analysis of the ADT‐Affected Metabolites. The top 50 enriched metabolic pathways affected by ADT over 6 months treatment course

Since the above findings indicate several ADT‐altered metabolites belong to similar metabolic pathways, we used MetaboAnalyst 4.0^24^ to identify the metabolic pathways in which multiple metabolites were significantly impacted by ADT. Instead of evaluating individual metabolite for the significance of their changes in response to ADT, the Metabolite Set Enrichment Analysis (MSEA)^25^ directly evaluated a set of functionally related metabolites for their functional enrichment. MSEA is based on a library of 1000 predefined metabolite sets covering various metabolic pathways, enabling the identification of obvious as well as “subtle but coordinated” changes among a group of related metabolites that may go undetected with conventional approaches. Therefore, similar to gene set enrichment analysis (GSEA),^26^ MSEA has the potential to identify subtle but consistent changes among a group of related compounds in the same metabolic pathways, which may go undetected with the conventional approaches. The top enriched pathways identified by MSEA in this study include steroid biosynthesis, phenylacetate metabolism, thiamine metabolism and pentose phosphate, mitochondria β‐oxidation, inositol phosphate metabolisms, pantothenate and CoA biosynthesis, and fatty acid metabolism (Figure 5). While some of these pathways were uncovered by the analysis of individual metabolites (steroid biosynthesis and fatty acid metabolism), MSEA also uncovered several metabolic pathways (thiamine metabolism and pentose phosphate, mitochondria β‐oxidation, inositol phosphate metabolisms, pantothenate and CoA biosynthesis^27^).

### 3.6 Correlation of blood chemistry with the ADT‐induced changes in androsterone sulfate

**Figure 6:**
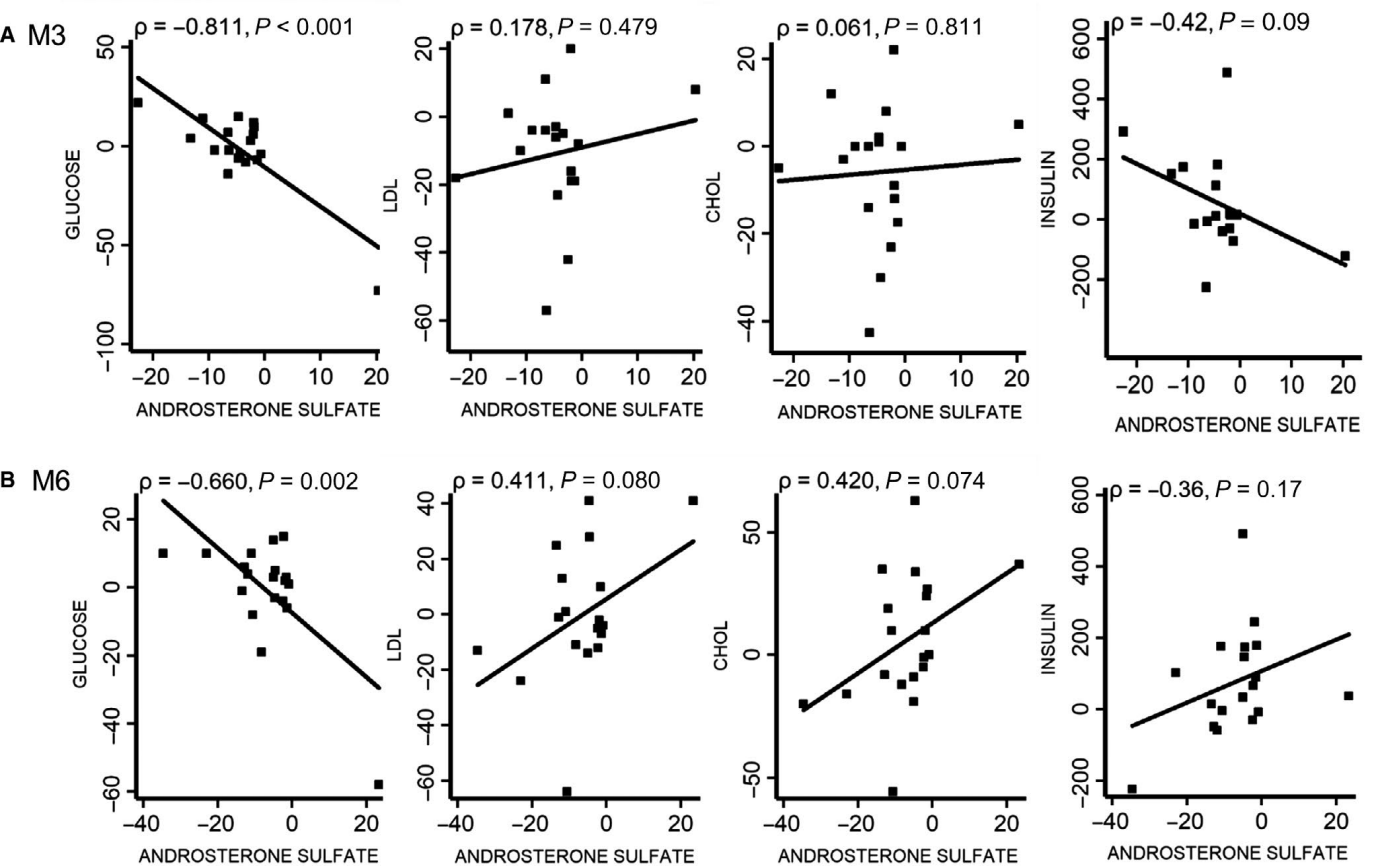
Serum Chemistry Correlated with ADT‐induced Changes in Androsterone Sulfate. The correlation between the ADT‐induced changes in the androsterone sulfate and serum glucose and lipids (A, B) The correlation between the ADT‐induced changes of the androsterone sulfate (reflecting the degree of ADT) and the changes in serum glucose, LDL, and cholesterol (CHOL) after 3 (A) and 6 (B) months of ADT

Given the top pathway of steroid biosynthesis is the intended targets of ADT, the reduced androsterone sulfate may serve as the direct on‐target metabolic endpoint to indicate the success of ADT to reduce the serum androgens. Therefore, we calculated the ADT‐induced changes in androsterone sulfate for each subject and then correlated the changes with various clinical laboratory parameters affected by ADT (Figure 6). We found that the degree of the androsterone sulfate was highly correlated with the changes in glucose at both 3 (*P* < .001) and 6 months (*P* = .002). The stronger repression of the ADT‐induced androsterone sulfate, the greater the elevation of the serum glucose in a dose‐response manner. In contrast, the changes in androsterone sulfate were not significantly correlated with the changes in insulin, LDL or total cholesterol. But this lack of correlation may be due to the small cohort size (20 individuals) in this study. While statistically insignificantly, the relationship between the androsterone sulfate and insulin was negative at M3 (Figure 6A), and then shifted to positive at M6 (Figure 6B). Neither correlation reached statistical significance. However, one possible explanation for the changes is the loss of muscle mass which is closely associated with insulin sensitivity. There seemed to be a tendency for the greater repression in ADT to be correlated with a greater rise in insulin at 3 months but this tendency disappeared at 6 months. In addition, at 6 months, the greater the repression in ADT seemed to be correlated with a tendency of lower LDL (*P* = .080) and total cholesterol (*P* = .074) but neither achieved statistical significance.

### 3.7 Identify metabolites whose changes correlate with androsterone sulfate

**Figure 7:**
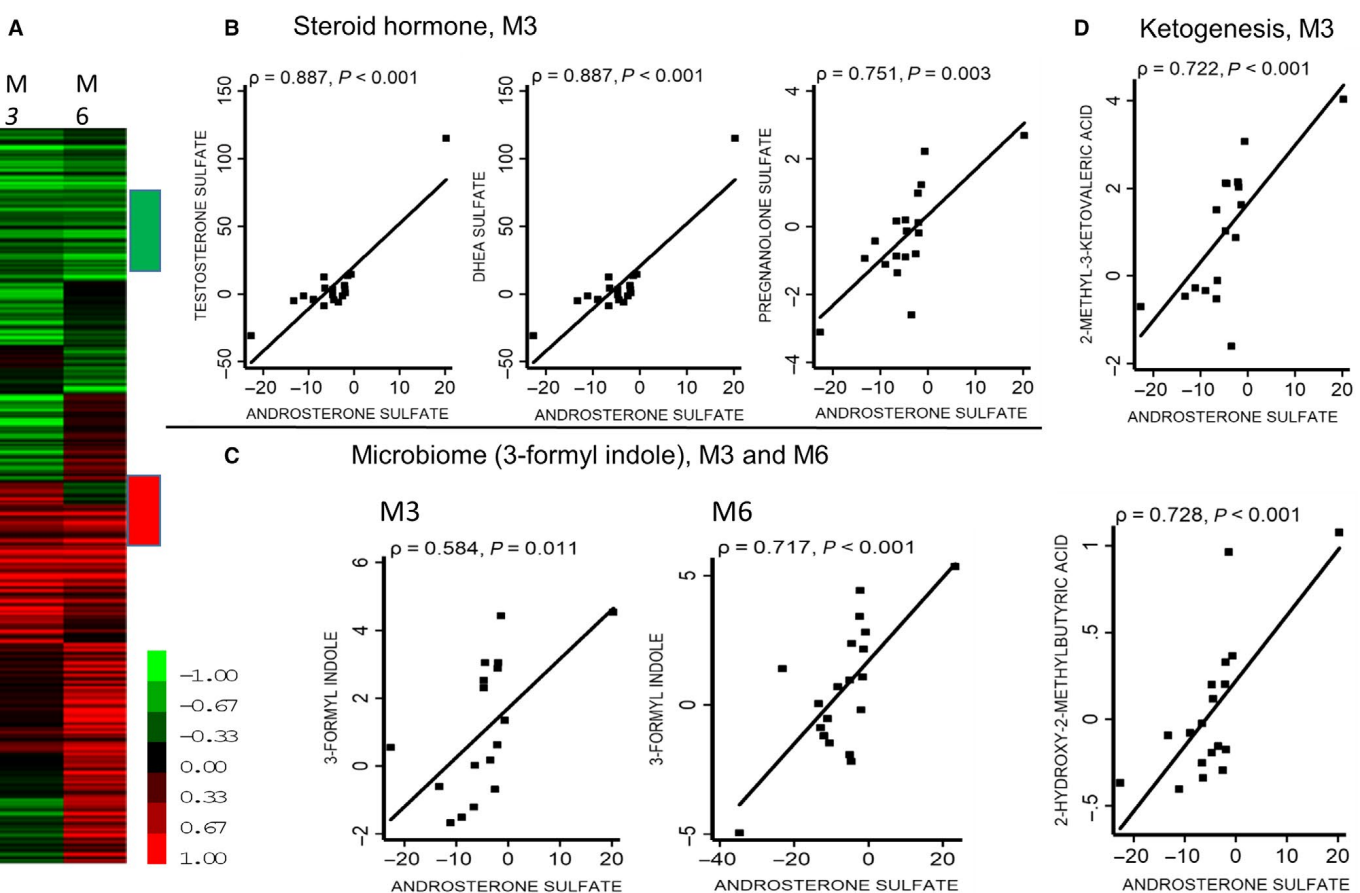
Metabolites Correlated with ADT‐induced Changes in Androsterone Sulfate. (A) Heatmap of the correlation between the ADT‐induced changes in metabolites with androsterone sulfate. Red: Positive correlation and Green: Negative correlation. (B‐D) Highly correlated associations between (B) steroid hormones, (C) microbiome metabolites (3‐formyl indole at 3 and 6 months), and (D) two ketogenesis metabolites with the ADT‐induced changes in androsterone sulfate

Figure 7 shows the significant correlation between ADT‐induced changes in androsterone sulfate with the changes in selected metabolites at 3 and 6 months. The correlation coefficient of the metabolites with androsterone sulfate were arranged by hierarchical clustering in a heatmap (Figure 7A, red: positive correlation, green: negative correlation with changes in androsterone sulfate). This analysis identified the clusters of metabolites whose changes were highly correlated with the changes in androsterone sulfate, either congruent (change in the same direction, red) or opposite (changes in the opposite direction, green). Among the congruent metabolites, we noted unsurprisingly several related male hormones, including testosterone sulfate, DHEA sulfate, and pregnanolone sulfate that were highly correlated with ADT‐induced changes in androsterone sulfate (Figure 7B). In addition, changes in androsterone sulfate were significantly correlated with changes in 3 Formyl‐indole (also known as Indole‐3‐carboxaldehyde (I3A) or indole‐3‐aldehyde), a metabolite of dietary L‐tryptophan that was synthesized by the gastrointestinal bacteria (Figure 7C) at both 3 month (*P* = .011) and 6 month (*P* < .001). Changes in androsterone sulfate were also significantly correlated with two metabolites of the ketogenesis pathway, 1 methyl‐3‐ketovaleric acid and 2‐hydroxy‐2‐methylbutyric acid (Figure 7D) after 3 months of ADT (both *P* < .001).

## 4 DISCUSSION

In this study, we applied MS‐based metabolomic profiling to determine the effects of 3 and 6 months of ADT on the serum metabolome. The key metabolic changes observed as a result of ADT can be summarized into at least four areas: (a) steroid metabolism, (b) ketogenesis, (c) fatty acid metabolism, and (d) microbiome metabolism. These changes were observed while the participants maintained similar dietary intake and exercise pattern from baseline to end of the 6 months study.^19^ As previously published, the control participants increased the median intake of carbohydrate and fat by about 10 grams/day and decreased protein and calories by about 6 grams/day and 95 kcal/day, respectively. In addition, these participants remained at similar exercise level from baseline to 6 months as reflected by the increase of 1 unit in MET score. Some of our findings are consistent with a previous study of 3 month ADT^18^ and additionally shows that the impact extends to 6 months of ADT treatment. In addition, our cohorts include 6 month and we applied some distinct analytic approaches and identified several new metabolomic responses and correlated these changes with blood chemistry and the changes of all metabolites to identify the changes which are correlated with the degrees of ADT‐induced changes in androgen sulfate. However, it is important to point out that this is a small cohort of 20 individuals. Therefore, there are significant limitations in the findings from such a small cohort and any findings need to be validated in much larger studies in the future. Furthermore, it will be clinically relevant to determine whether different forms of ADT (eg, GnRH agonist, antagonist, or surgery) would have different metabolic signatures. However, we would surmise that the dominant effect seen would be related to castration, regardless of how this was achieved. Unfortunately, these data were not captured in the trial. That being said, in clinical practice orchiectomy and GnRH antagonists are rarely used and thus it is likely nearly all or all the men in this study were GnRH agonist patients. Regardless, it will be important to consider identifying any treatment‐specific metabolic effects in the future studies.

The side effect of ADT has been shown previously to include metabolic disturbances such as impaired glucose tolerance and insulin resistance, which increase the risk for diabetes and CV death.^3^ Our data also support such an effect. We found that the ADT‐induced reduction in androgen sulfate is associated with an elevation in serum glucose and a greater rise in insulin at 3 months. At 6 months, the greater the ADT‐induced repression of androgen sulfate tends to be associated with a lower LDL (*P* = .080) and total cholesterol (*P* = .074). The significant correlation between the reduction in androsterone sulfate and rise in fasting glucose is consistent with the clinical evidence that ADT leads to increase risk of glucose intolerance and insulin resistance. However, the underlying mechanisms are unclear why the insulin level did not respond in tandem with glucose at both time points.

As expected from ADT, there was a significant reduction in the steroid biosynthesis as shown in the reduced androsterone sulfate, DHEA sulfate, and pregnanolone sulfate. This finding also objectively verifies the intended impact of the therapy to reduce various steroid species. In addition, we observed that 3‐hydroxybutyric acid was reduced at both 3 and 6 months. 3‐hydroxybutyric acid is a ketone body synthesized in the liver from acetyl‐CoA and can be used as an alternative energy source when blood glucose is low. This reduction of ketone body may be due to the increased blood glucose in response to ADT at both time points. The reduction of the 3‐hydroxybutyric acid indicates that ADT either reduced acetyl‐CoA/ketogenesis or increased the consumption of ketone bodies. Ketone pathway is found to be regulated by many oncogenic pathways.^28^, ^29^ Previous quantitative proteomics reveals that multiple ketogenic enzymes and increase in β‐hydroxybutyrate are associated with PC progression.^30^ Ketone body is also postulated to trigger insulin resistance and the ability of ketogenic diets to mitigate insulin resistance may be associated with the ability of ketone bodies to overcome insulin resistance.^31^ Therefore, the ADT‐associated reduced ketone body may contribute to the insulin resistance seen in these patients. Although speculative at this point, we are curious about the potential of ketogenic diets to prevent ADT‐induced insulin resistance, similar to our previous study of low carbohydrate diets.^19^

In addition to the impact on ketogenesis, there is a consistent reduction in various acyl‐carnitine and fatty acids metabolites including palmitoleic acid, octanoylcarnitine, palmitoylcarnitine indicating that ADT significantly reduced fatty acid β‐oxidation. Importantly, there was no change in the free carnitine. This finding is again similar to the previous study by Saylor et al^18^ but additionally demonstrates that the impact remains till 6 months of ADT. Acyl‐carnitines are generated by carnitine acyltransferases from combining carnitine and acyl‐CoAs as metabolic intermediates of fatty acid metabolism. The accumulation of acyl‐carnitines usually occurs when there is imbalance between β‐oxidation and the tricarboxylic acid (TCA) cycle. In some studies, the accumulated acyl‐carnitines in plasma are a transport form of various acyl‐groups between tissues and utilized for energy production in various tissues. High levels of acyl‐carnitines are also associated with diet‐induced obesity and insulin resistance in both animal models and human.^32^, ^33^ Insulin infusion directly reduced the level of long‐chain acyl‐carnitines in plasma. Therefore, elevated acylcarnitine have been postulated to contribute to insulin resistance phenotypes^34^ of ADT. In our study, we found that while increasing serum glucose, ADT consistently reduced the level of long‐chain acyl‐carnitines without affecting free carnitines. Therefore, it is reasonable to conclude that ADT impacted beta‐oxidation and resulted in the changes of acyl‐carnitines and ketone bodies in circulation. Such lower levels of acyl‐carnitines associated with insulin resistance was also noted among HIV+ individuals.^35^ Our results may suggest that long‐chain acyl‐carnitines may not be responsible for insulin resistance during ADT treatment and/or ADT‐insulin resistance occurs through distinct mechanisms which do not involve the elevated long‐chain acyl‐carnitines. Since acyl‐carnitines was found to be higher in PC,^36^ the reduced serum long‐chain acylcarnitine may reflect the therapeutic effects of ADT for PC and may serve as potential biomarkers for ADT response.

During oncogenesis, tumors with particular oncogenic mutations are found to develop addictions to particular sets of amino acids.^37^, ^38^, ^39^ Furthermore, the nutrient and metabolic status may also help to shape the genetic landscape of tumors.^40^, ^41^, ^42^ During the past decade, one of the consistent and reliable metabolic biomarkers for insulin resistance is the elevated branched‐chain amino acids (BCAAs).^43^ BCAA metabolic signature is also postulated to be an important contributor to the insulin resistance and targeting BCAA may have therapeutic potential.^44^ While ADT is known to be associated with insulin resistance, we did not notice an elevated BCAA metabolic signature. Similar lack of BCAA signature was also noted in the insulin resistance in HIV+ patients.^35^ The lack of BCAA metabolic signature may suggest that ADT‐induced insulin resistance may occur through a distinct metabolism, such as the reduced ketone bodies and insulin response. Our future analyses of men on the LCD may allow us to test this hypothesis.

Unexpectedly, we found that ADT reduces the level of Indole Acetic Acid (IAA), a breakdown product of tryptophan metabolism that is often produced by the action of bacteria in the mammalian gut. IAA is an agonist for the aryl hydrocarbon receptor in intestinal immune cells to stimulate the production of IL‐22 which facilitates the regulation of mucosal reactivity and inflammation.^23^ It is interesting to note that there are significant sex‐specific features of the microbiome that is partially driven by sex hormone.^45^, ^46^ Studies in murine models demonstrate that castration and antiandrogens can affect the composition of gut microbiota.^47^, ^48^ ADT is also associated with changes in the microbiomes.^49^, ^50^, ^51^ Furthermore, IL‐22 has been recently associated with the development of Polycystic ovary syndrome (PCOS),^52^ a disease characterized by androgen excess, accompanied by insulin resistance.^53^ Therefore, our observation may suggest a novel role of ADT‐induced microbiome, IAA and IL‐22 to exert metabolic influences on inflammation, which may contribute to the metabolic effects of ADT. In the future, simultaneous profiling of the microbiome and gut metabolisms during ADT may help to elucidate the role of microbiome in the treatment efficacy and side effects of ADT.

## CONFLICT OF INTEREST

No conflict of Interests.

## AUTHOR CONTRIBUTIONS

JTC: data analysis, manuscript writing – original draft, review, and editing. PHL: Conceptualization, data analysis, manuscript writing. VT, VB, BG, RS: methodology, data acquisition, statistical analysis. TO: statistical analysis. EC: methodology, project administration, data acquisition, statistical analysis. MAK: Conceptualization, methodology, project administration, data acquisition, statistical analysis. SJF: Conceptualization, funding acquisitions, manuscript writing.

## Supporting information

Figure S1

Table S1
Table S2

## Data Availability

The metabolomic data will be made available to the academic community upon publication of the manuscript.
