# Supplementary figures and images for: Metabolomic effects of androgen deprivation therapy treatment for prostate cancer
Source: Cancer Med. 2020 Mar 31;9(11):3691–702. doi: 10.1002/cam4.3016 (PMC7286468; doi:10.1002/cam4.3016)

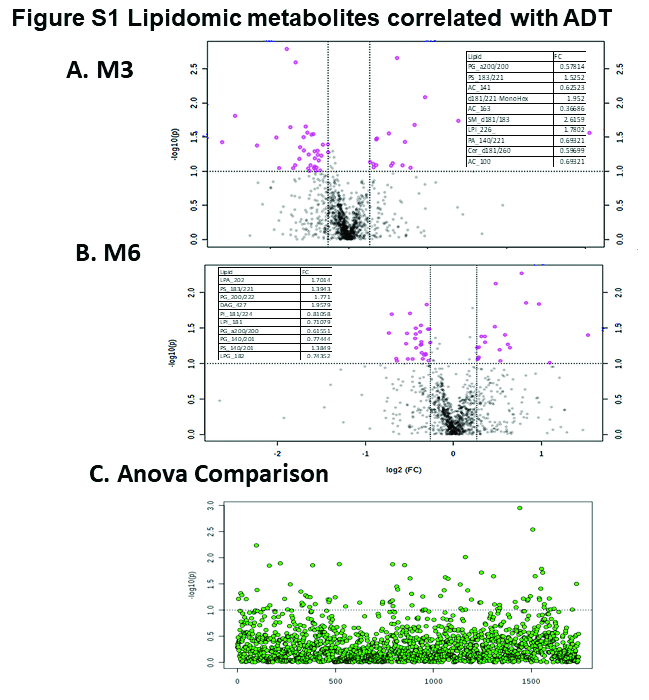

Supplement: Supplementary file 1 — Figure S1 [file CAM4-9-3691-s001.tif]
