# Supplementary material for: Metabolomic effects of androgen deprivation therapy treatment for prostate cancer
Source: Cancer Med. 2020 Mar 31;9(11):3691–702. doi: 10.1002/cam4.3016 (PMC7286468; doi:10.1002/cam4.3016)
Supplement: Supplementary file 2 — Table S1 Table S2 [file CAM4-9-3691-s002.docx]

|  | **Table S1: Top 50 lipidomic features at 3 months** | | | |  |
| --- | --- | --- | --- | --- | --- |
|  | Compounds | FC | log2(FC) | raw.pval | "-log10(p)" |
| 1 | PGa200/200 | 0.57814 | -0.79051 | 0.001612 | 2.7926 |
| 2 | PS183/221 | 1.5252 | 0.60902 | 0.0021904 | 2.6595 |
| 3 | AC141 | 0.62523 | -0.67755 | 0.0025358 | 2.5959 |
| 4 | d181/221-MonoHex | 1.952 | 0.96497 | 0.0082303 | 2.0846 |
| 5 | AC163 | 0.36686 | -1.4467 | 0.015405 | 1.8123 |
| 6 | SMd181/183 | 2.6159 | 1.3873 | 0.018248 | 1.7388 |
| 7 | LPI226 | 1.7802 | 0.83203 | 0.020928 | 1.6793 |
| 8 | PA140/221 | 0.68316 | -0.54971 | 0.022115 | 1.6553 |
| 9 | Cerd181/260 | 0.59699 | -0.74421 | 0.022617 | 1.6456 |
| 10 | AC100 | 0.69321 | -0.52864 | 0.027215 | 1.5652 |
| 11 | SMd181/140 | 8.2927 | 3.0518 | 0.02744 | 1.5616 |
| 12 | SMd161/160 | 1.42 | 0.5059 | 0.027937 | 1.5538 |
| 13 | TAG586NH4 | 0.7307 | -0.45265 | 0.028562 | 1.5442 |
| 14 | PG120/120 | 0.71862 | -0.47669 | 0.029036 | 1.5371 |
| 15 | d181/226-DiHex | 0.67059 | -0.57649 | 0.03166 | 1.4995 |
| 16 | Cerd182/200 | 0.52752 | -0.9227 | 0.032036 | 1.4944 |
| 17 | d181/140-DiHex | 1.277 | 0.35281 | 0.033011 | 1.4813 |
| 18 | PI183/202 | 1.2708 | 0.34579 | 0.034277 | 1.465 |
| 19 | LPI202 | 1.6355 | 0.70974 | 0.037182 | 1.4297 |
| 20 | SMd181/141 | 0.32755 | -1.6102 | 0.037487 | 1.4261 |
| 21 | PI181/224 | 0.83075 | -0.26751 | 0.040407 | 1.3935 |
| 22 | DAG401NH4 | 0.79435 | -0.33216 | 0.040838 | 1.3889 |
| 23 | LPS203 | 0.44508 | -1.1679 | 0.041931 | 1.3775 |
| 24 | CE242NH4 | 0.65034 | -0.62074 | 0.044507 | 1.3516 |
| 25 | d181/202-DiHex | 0.67182 | -0.57385 | 0.049595 | 1.3046 |
| 26 | PI140/222 | 0.75791 | -0.3999 | 0.050073 | 1.3004 |
| 27 | PIp160/222 | 0.73919 | -0.43598 | 0.051125 | 1.2914 |
| 28 | PI181/201 | 0.8315 | -0.26622 | 0.05275 | 1.2778 |
| 29 | PS161/201 | 0.7014 | -0.5117 | 0.056855 | 1.2452 |
| 30 | LPG161 | 0.75963 | -0.39663 | 0.057437 | 1.2408 |
| 31 | AC101 | 0.78388 | -0.3513 | 0.059114 | 1.2283 |
| 32 | PGp200/161 | 0.74065 | -0.43313 | 0.064669 | 1.1893 |
| 33 | d181/220-DiHex | 0.6461 | -0.63017 | 0.065919 | 1.181 |
| 34 | TAG629NH4 | 0.76598 | -0.38463 | 0.06947 | 1.1582 |
| 35 | PG203/223 | 1.203 | 0.26661 | 0.072868 | 1.1375 |
| 36 | LPA202 | 1.4677 | 0.5536 | 0.07639 | 1.117 |
| 37 | PE381 | 1.237 | 0.30684 | 0.078124 | 1.1072 |
| 38 | LPG120 | 1.2692 | 0.34388 | 0.081458 | 1.0891 |
| 39 | PG222/226 | 0.71268 | -0.48867 | 0.081779 | 1.0874 |
| 40 | AC140 | 0.62221 | -0.68452 | 0.081791 | 1.0873 |
| 41 | CE243NH4 | 1.6008 | 0.67877 | 0.08202 | 1.0861 |
| 42 | d181/241-TriHex | 1.4481 | 0.53421 | 0.082816 | 1.0819 |
| 43 | d182/141-MonoHex | 0.73913 | -0.4361 | 0.085535 | 1.0679 |
| 44 | PG200/202 | 1.242 | 0.31267 | 0.08758 | 1.0576 |
| 45 | PEO-363 | 0.7516 | -0.41196 | 0.088578 | 1.0527 |
| 46 | PG200/222 | 1.718 | 0.7807 | 0.088845 | 1.0514 |
| 47 | PEO-364 | 0.73436 | -0.44545 | 0.089662 | 1.0474 |
| 48 | LPI201 | 0.54085 | -0.8867 | 0.090042 | 1.0456 |
| 49 | Cerd181/221 | 0.61151 | -0.70956 | 0.090163 | 1.045 |
| 50 | PAp180/181 | 0.69852 | -0.51763 | 0.091166 | 1.0402 |

|  | **Table S2: Top lipidomic features at 6 months by volcano plot** | | | | |
| --- | --- | --- | --- | --- | --- |
|  | Compounds | FC | log2(FC) | raw.pval | "-log10(p)" |
| 1 | LPA202 | 1.7014 | 0.76676 | 0.0050429 | 2.2973 |
| 2 | PS183/221 | 1.3943 | 0.47958 | 0.0074506 | 2.1278 |
| 3 | PG200/222 | 1.771 | 0.82454 | 0.013928 | 1.8561 |
| 4 | DAG427NH4 | 1.9597 | 0.97065 | 0.014465 | 1.8397 |
| 5 | PI181/224 | 0.81058 | -0.30298 | 0.014723 | 1.832 |
| 6 | LPI181 | 0.71079 | -0.49251 | 0.019603 | 1.7077 |
| 7 | PGa200/200 | 0.61551 | -0.70014 | 0.020109 | 1.6966 |
| 8 | PG140/201 | 0.77444 | -0.36878 | 0.028904 | 1.539 |
| 9 | PS161/203 | 1.3849 | 0.46976 | 0.030137 | 1.5209 |
| 10 | LPG182 | 0.74352 | -0.42755 | 0.031695 | 1.499 |
| 11 | PI181/201 | 0.81848 | -0.28898 | 0.032607 | 1.4867 |
| 12 | PI182/223 | 0.82807 | -0.27218 | 0.032641 | 1.4862 |
| 13 | PG120/120 | 0.76914 | -0.37869 | 0.035695 | 1.4474 |
| 14 | AC141 | 0.60203 | -0.73208 | 0.037089 | 1.4307 |
| 15 | Cerd181/221 | 0.69392 | -0.52716 | 0.03754 | 1.4255 |
| 16 | PI181/200 | 0.74357 | -0.42746 | 0.038248 | 1.4174 |
| 17 | PS201/203 | 1.5005 | 0.58547 | 0.039352 | 1.405 |
| 18 | DAG420NH4 | 2.8817 | 1.5269 | 0.039605 | 1.4022 |
| 19 | PI140/221 | 1.2465 | 0.31786 | 0.041353 | 1.3835 |
| 20 | PC449/PCO-442 | 1.2808 | 0.35704 | 0.041405 | 1.3829 |
| 21 | LPG181 | 0.73993 | -0.43453 | 0.044179 | 1.3548 |
| 22 | PI182/200 | 0.77711 | -0.36381 | 0.049329 | 1.3069 |
| 23 | PC448/PCO-441 | 1.2821 | 0.35848 | 0.049987 | 1.3011 |
| 24 | PG181/201 | 0.83282 | -0.26392 | 0.050792 | 1.2942 |
| 25 | AC163 | 0.68928 | -0.53684 | 0.052396 | 1.2807 |
| 26 | DAG300NH4 | 1.5341 | 0.61742 | 0.053547 | 1.2713 |
| 27 | PI181/221 | 0.77784 | -0.36245 | 0.053569 | 1.2711 |
| 28 | LPG201 | 0.77262 | -0.37216 | 0.054459 | 1.2639 |
| 29 | PG140/205 | 1.2218 | 0.28899 | 0.058633 | 1.2319 |
| 30 | LPI226 | 1.2047 | 0.26867 | 0.059512 | 1.2254 |
| 31 | CE120NH4 | 1.5596 | 0.64117 | 0.059538 | 1.2252 |
| 32 | AC180 | 0.74633 | -0.42212 | 0.059847 | 1.223 |
| 33 | SMd161/200 | 1.4381 | 0.52421 | 0.063768 | 1.1954 |
| 34 | d181/160-DiHex | 0.77947 | -0.35944 | 0.070316 | 1.1529 |
| 35 | PGa160/140 | 0.80278 | -0.31692 | 0.072257 | 1.1411 |
| 36 | PI183/221 | 0.80411 | -0.31454 | 0.075105 | 1.1243 |
| 37 | d181/160-MonoHex | 0.79289 | -0.33481 | 0.075783 | 1.1204 |
| 38 | PG183/205 | 1.2204 | 0.28734 | 0.081188 | 1.0905 |
| 39 | PG203/223 | 1.2153 | 0.2813 | 0.08438 | 1.0738 |
| 40 | LPE161 | 0.63808 | -0.6482 | 0.084903 | 1.0711 |
| 41 | LPE204 | 0.78575 | -0.34785 | 0.08549 | 1.0681 |
| 42 | PI140/222 | 0.69888 | -0.51689 | 0.085959 | 1.0657 |
| 43 | PIp200/183 | 0.72536 | -0.46323 | 0.086061 | 1.0652 |
| 44 | PG202/224 | 1.2092 | 0.27409 | 0.086889 | 1.061 |
| 45 | PI201/204 | 0.82228 | -0.2823 | 0.090529 | 1.0432 |
| 46 | PG203/222 | 1.4468 | 0.53285 | 0.091455 | 1.0388 |
| 47 | LPE180 | 0.64085 | -0.64193 | 0.091611 | 1.0381 |
| 48 | TAG508NH4 | 2.1324 | 1.0925 | 0.09707 | 1.0129 |
